# Catatonia with Psychosis in an 8-Year-Old Child: A Case Report and a Literature Review

**DOI:** 10.1155/2022/4124733

**Published:** 2022-03-25

**Authors:** Margaret D. Weiss, Larry Schibuk, Srinivasa B. Gokarakonda, Renea Henderson, Dianna Esmaeilpour

**Affiliations:** ^1^Child Inpatient in the Department of Child Psychiatry, Cambridge Health Alliance, 1493 Cambridge St., Cambridge, MA 02138, USA; ^2^Lahey Hospital and Medical Center, Burlington, MA, USA; ^3^Department of Child Psychiatry, University of Arkansas Medical Sciences, Little Rock AR 72209, USA; ^4^Chenal Family Therapy, Rogers, AR, USA; ^5^Private practice psychiatry, Bentonville, AR, USA

## Abstract

**Objective:**

We present a narrative review of pediatric catatonia and a case report illustrating the complexity of management of psychosis in a child with catatonia.

**Method:**

The literature search used the text terms pediatric, catatonia, and antipsychotics and the search engines PubMed and EBSCO. All references from peer-reviewed journals were reviewed for treatment strategies specific to management in children who are also psychotic. *Findings*. This 8-year-old girl presented with psychotic symptoms which were initially treated with antipsychotics and evolved into life-threatening catatonia that was eventually stabilized with a total daily dose of 46 mg of lorazepam. Lower doses led to recurrence. Once catatonia improved, she tolerated combined benzodiazepine and antipsychotic treatment. Long-term maintenance over 5 years required maintenance treatment with both benzodiazepines and antipsychotics to prevent relapse.

**Conclusions:**

The extraordinary doses of benzodiazepines found to be optimal for management of catatonia in this child led to improved alertness and orientation, without evident sedation. Catatonia did not recur with later management of psychosis using neuroleptics when added to lorazepam. The current literature on pediatric catatonia does not provide guidance on dose maintenance or when and if to rechallenge with antipsychotics.

## 1. Introduction

Catatonia is considered a unique neurobiological syndrome [1], described in *DSM 5* as including three diagnostic categories: catatonic disorder due to another medical condition, catatonia associated with another mental disorder (catatonia specifier), and unspecified catatonia. The traditional category of catatonic schizophrenia has been deleted. The *DSM 5* criteria require three of the following symptoms: stupor, catalepsy, waxy flexibility, mutism, negativism, posturing, mannerisms, stereotypy, agitation, grimacing, echolalia, and echopraxia. Rigidity, self-injurious behavior, and repetitive movements have also been described [2].

Catatonia is considered to be “a systemic medical syndrome” [3, 4] with a prevalence of approximately 7% [5]–10% [6, 7] of inpatient admissions and a 60-fold increase in mortality as compared with youths in the general population [8] or up to 10–20% of cases [2]. The prevalence of catatonia in autism may be as high as 17% [9] and more likely to become recurrent or chronic [10]. Catatonia can be retarded and/or excited and in its most severe form is known as “malignant” catatonia with delirium, fever, autonomic instability, and motoric abnormalities. An in vivo single-photon emission computed tomography (SPECT) study of benzodiazepine distribution catatonic symptoms showed a decreased density of gamma-aminobutyric acid-A (GABA-A) in the left sensorimotor and right parietal cortex [11]. A functional magnetic resonance imaging (fMRI) study showed reduced motor activation of the contralateral motor cortex [12]. The patient was never able to comply with or consent to getting an electroencephalogram (EEG). Malignant catatonia is an acute toxic state that can be precipitated by the use of antipsychotics [13–16].

A wide range of both psychiatric and medical conditions have been found to be associated with catatonia including autism [9, 17–21], schizophrenia [22, 23], trauma [24, 25], affective disorders [8], Pediatric Acute-Onset Neuropsychiatric Syndrome (PANS) [26], Tourette syndrome, mutism, and obsessive-compulsive disorder (OCD) [27]. Medical conditions associated with catatonia have included anti-N-methyl-D-aspartate (anti-NMDA) encephalitis and other forms of autoimmune encephalitis [28–33], autoimmune disorders [34], systemic lupus erythematosus [35, 36], encephalitis [37], infection [38], seizures [39], drug reactions [40–43], genetic disorders [44], and inborn errors of metabolism [39]. Dhossche and Wachtel reported on a host of other pediatric disorders that can present with catatonia, leading him to refer to the disorder as “hidden in plain sight” (1). Lack of recognition of the disorder has been a particular concern, especially in view of the concern that misdiagnosis and treatment with neuroleptics could lead to a worsening of the condition (2).

Lorazepam [45–47] and electroconvulsive therapy (ECT) [45–59] have been described in multiple case reports as effective treatments. Lorazepam has been used both as a challenging procedure to facilitate the diagnosis of catatonia [60] and as treatment with a range from low [46] to very high oral or parenteral [61] doses. Difficulties with consenting and access to ECT in pediatrics have meant that ECT has been used largely in cases refractory to lorazepam, although ECT has been shown to be quick and effective [54, 62].

Most of what we know about catatonia has been based on case series, in which expert consensus or historical precedent has been the basis for treatment recommendations. In a review of the literature and case reports, we found a lack of clarity on benzodiazepine dose, adverse events associated with benzodiazepine treatment, required duration of benzodiazepine treatment, potential problems in tapering lorazepam, and challenges in outpatient use of high-dose lorazepam.

Our review did not identify any guidance on the use of neuroleptics in children with catatonia and psychosis. Of the many cases of catatonia reported in the literature, we only found four reports in which treatment included both lorazepam and an antipsychotic (olanzapine) [63–66], although this does not mean combination antipsychotic/lorazepam treatment is not occurring in practice. If antipsychotics are considered to be contraindicated in catatonia, this raises the question of how to treat chronic or recurrent catatonia in the presence of serious psychosis, especially when ECT is not an option.

We present a case of life-threatening catatonia in an 8-year-old child. The case is remarkable for the young age of presentation, the presence of ongoing psychosis, the need for extreme doses of benzodiazepines to stabilize the catatonia, and tolerance of antipsychotic medication when added to lorazepam, despite ongoing symptoms of catatonia. All identifying details have been removed, and the IRB has approved publication without patient consent.

## 2. Case Report

This was an immigrant Marshallese family living in a rural area. Past development included developmental and language delays. The only family psychiatric history was a one-week episode of sleeplessness, agitation, odd behavior, and fearfulness in an older sister at age 3 years.

At age 8 years, the patient presented with a new onset of isolation, fearfulness, mumbling and laughing to herself, sleeplessness, and screaming. She was observed to be hallucinating and referred to herself by a name other than her own. Teachers complained of threatening behavior and aggression. The emergency psychiatrist diagnosed childhood-onset psychosis and started oral aripiprazole 5 mg daily, which was ineffective, and switched to oral risperidone 1 mg. There was no rigidity, fever, or abnormality in vital signs. She received IV hydration and lorazepam 1.5 mg and midazolam 3 mg once and was transferred to a tertiary children's hospital pediatric intensive care unit in restraints because of extreme excitement, food and liquid refusal, absence of sleep, negativism, grimacing, and self-injurious behavior. Physical exam remained normal. She was diagnosed as having excited catatonia. She was unable to communicate, incontinent, and unresponsive to painful stimuli.

An organic workup including comprehensive lab work, MRI, cerebrospinal fluid (CSF) studies, anti-NMDA antibodies, heavy metal and copper testing, autoimmune studies, and antistreptolysin O (ASO) titers was unremarkable. Antipsychotics were discontinued and sedation initiated with dexmedetomidine (an alpha 2 adrenergic agonist) at 0.4 mcg/g/h and lorazepam 2 mg IV q6hrs. The lorazepam dose was increased and dexmedetomidine discontinued over three days. The patient was transferred from the intensive care unit (ICU) to a pediatric ward on midazolam at 0.1 mg/kg/hr 2.5 mg q4 hrs and lorazepam 2.5 mg q4H with additional PRN doses for agitation. IV medication was gradually switched to oral lorazepam, which had to be titrated to 9 mg po qid or 36 mg total daily dose. In addition to the scheduled lorazepam, the patient received prn lorazepam, such that the highest total daily dose of lorazepam administered was 46 mg. This dose of lorazepam was tolerated without sedation, respiratory difficulty, slurred speech, or ataxia. Despite improvement, the patient was still hallucinating.

The patient was admitted to psychiatry inpatient for management of psychosis on lorazepam which was cross-tapered over 3 weeks to clonazepam 1 mg qam and 2 mg po qhs. After an eight-week inpatient psychiatry stay, she was discharged on this dose of clonazepam and risperidone 1 mg PO QAM and 2 mg PO QHS. The antipsychotic was changed to aripiprazole 2 mg po qd at the patient's request, and clonazepam was maintained at 2 mg qd. Episodes of accidental abrupt discontinuation of clonazepam would lead to an acute deterioration with expected withdrawal symptoms such as insomnia and also other symptoms consistent with the initial presentation of catatonia such as extreme excitement and screaming. Episodic attempts to reduce the dose of benzodiazepine would also lead to a recurrence of catatonic symptoms. Five years later, she remains stable on aripiprazole 2 mg and clonazepam 2 mg.

## 3. Discussion

The presentation of excited catatonia in this child is consistent with past literature in the acute onset, extreme fear, absence of medical findings, and the response to high doses of lorazepam. However, several aspects of the presentation are unique. This is the youngest case of catatonia we identified in the literature. The dose of lorazepam required to stabilize the catatonia was considerably higher than has been previously reported and seemed to be tolerated without any of the usual expected side effects of benzodiazepines and a clearing of sensorium rather than confusion. This raises the possibility that high-dose benzodiazepine treatment in catatonia has a distinct pathway of action, as opposed to benzodiazepine treatment of anxiety, perhaps in remediating the GABA-A abnormalities associated with the disorder. There are 4 previous case reports of treatment of catatonia and psychosis, in which psychosis has been successfully treated with antipsychotic medication following stabilization with benzodiazepine [63–66]. Our case presentation provides support for the conclusion of one of these authors who proposed, “a modification to the standard treatment protocol for catatonia, especially in those patients with schizophrenia with catatonic features” to consider the introduction of antipsychotic treatment earlier in treatment [63]. It is noteworthy in this case that antipsychotic medication was poorly tolerated and ineffective, until after the patient was stabilized on lorazepam. Maintenance treatment was required for both catatonia and psychosis which had a chronic course without return to the previous level of functioning. Maintenance outpatient treatment of benzodiazepines in a young child also raises concerns, such as the risk of a seizure in the event of nonadherence. This single case report of management of catatonia and psychosis in a young child contributes to the literature on this unique comorbidity. It is limited by the absence of sequential ratings on the Busch Francis Rating Scale, although this scale is not validated beyond agreement with clinical observation which is well described in the case report [5] and also not validated in young children. A case series looking at alternative treatment strategies and outcomes for catatonia in young children with psychosis is needed. Catatonia presenting with psychosis may require aggressive treatment of both conditions.

## References

[1] Dhossche D. M., Wachtel L. E. (2010). Catatonia is hidden in plain sight among different pediatric disorders: a review article. *Pediatric Neurology*.

[2] Ghaziuddin N., Wachtel L. (2019). Catatonia: treatment with a benzodiazepine. *AACAP News*.

[3] Fink M., Fricchione G., Rummans T., Shorter E. (2016). Catatonia is a systemic medical syndrome. *Acta Psychiatrica Scandinavica*.

[4] Fink M., Shorter E., Taylor M. A. (2010). Catatonia is not schizophrenia: Kraepelin's error and the need to recognize catatonia as an independent syndrome in medical nomenclature. *Schizophrenia Bulletin*.

[5] Bush G., Fink M., Petrides G., Dowling F., Francis A. (1996). Catatonia. I. Rating scale and standardized examination. *Acta Psychiatrica Scandinavica*.

[6] Taylor M. A., Fink M. (2003). Catatonia in psychiatric classification: a home of its own. *The American Journal of Psychiatry*.

[7] Subramaniyam B. A., Muliyala K. P., Hari Hara S., Kumar Reddi V. S. (2019). Prevalence of catatonic signs and symptoms in an acute psychiatric unit from a tertiary psychiatric center in India. *Asian Journal of Psychiatry*.

[8] Cornic F., Consoli A., Tanguy M. L. (2009). Association of adolescent catatonia with increased mortality and morbidity: evidence from a prospective follow-up study. *Schizophrenia Research*.

[9] Wing L., Shah A. (2006). A systematic examination of catatonia‐like clinical pictures in autism spectrum disorders. *International Review of Neurobiology*.

[10] Barnes M. P., Saunders M., Walls T. J., Saunders I., Kirk C. A. (1986). The syndrome of Karl Ludwig Kahlbaum. *Journal of Neurology, Neurosurgery, and Psychiatry*.

[11] Northoff G., Steinke R., Czcervenka C. (1999). Decreased density of GABA-A receptors in the left sensorimotor cortex in akinetic catatonia: investigation of in vivo benzodiazepine receptor binding. *Journal of Neurology, Neurosurgery, and Psychiatry*.

[12] Northoff G., Braus D. F., Sartorius A. (1999). Reduced activation and altered laterality in two neuroleptic-naive catatonic patients during a motor task in functional MRI. *Psychological Medicine*.

[13] Casey D. A. (1987). Electroconvulsive therapy in the neuroleptic malignant syndrome. *Convulsive Therapy*.

[14] Cohen S., Fligner C. L., Raisys V. A., Luthi R., Dunner D. L. (1987). A case of nonneuroleptic malignant syndrome. *The Journal of Clinical Psychiatry*.

[15] Lee J. W. (2000). Catatonic and non-catatonic neuroleptic malignant syndrome. *The Australian and New Zealand Journal of Psychiatry*.

[16] Northoff G. (2002). Catatonia and neuroleptic malignant syndrome: psychopathology and pathophysiology. *Journal of Neural Transmission (Vienna)*.

[17] Bozkurt H., Mukaddes N. M. (2010). Catatonia in a child with autistic disorder. *The Turkish Journal of Pediatrics*.

[18] Dhossche D. M., Shah A., Wing L. (2006). Blueprints for the assessment, treatment, and future study of catatonia in autism spectrum disorders. *International Review of Neurobiology*.

[19] Guinchat V., Cravero C., Diaz L. (2015). Acute behavioral crises in psychiatric inpatients with autism spectrum disorder (ASD): recognition of concomitant medical or non-ASD psychiatric conditions predicts enhanced improvement. *Research in Developmental Disabilities*.

[20] Wing L., Shah A. (2000). Catatonia in autistic spectrum disorders. *The British Journal of Psychiatry*.

[21] DeJong H., Bunton P., Hare D. J. (2014). A systematic review of interventions used to treat catatonic symptoms in people with autistic spectrum disorders. *Journal of Autism and Developmental Disorders*.

[22] Bonnot O., Tanguy M. L., Consoli A. (2008). Does catatonia influence the phenomenology of childhood onset schizophrenia beyond motor symptoms?. *Psychiatry Research*.

[23] Cohen D., Nicolas J. D., Flament M. F. (2005). Clinical relevance of chronic catatonic schizophrenia in children and adolescents: evidence from a prospective naturalistic study. *Schizophrenia Research*.

[24] Benarous X., Raffin M., Bodeau N., Dhossche D., Cohen D., Consoli A. (2017). Adverse childhood experiences among inpatient youths with severe and early-onset psychiatric disorders: prevalence and clinical correlates. *Child Psychiatry and Human Development*.

[25] Dhossche D. M., Ross C. A., Stoppelbein L. (2012). The role of deprivation, abuse, and trauma in pediatric catatonia without a clear medical cause. *Acta Psychiatrica Scandinavica*.

[26] Schlansky K., Facer B., Tanguturi Y. C., Cundiff A. W., Fuchs D. C. (2020). Pediatric acute-onset neuropsychiatric syndrome and catatonia: a case report. *Psychosomatics*.

[27] Fink M. (2013). Rediscovering catatonia: the biography of a treatable syndrome. *Acta Psychiatrica Scandinavica. Supplementum*.

[28] Abe K. K., Koli R. L., Yamamoto L. G. (2016). Emergency department presentations of anti-N-methyl-D-aspartate receptor encephalitis. *Pediatric Emergency Care*.

[29] Consoli A., Ronen K., An-Gourfinkel I. (2011). Malignant catatonia due to anti-NMDA-receptor encephalitis in a 17-year-old girl: case report. *Child and Adolescent Psychiatry and Mental Health*.

[30] Dhossche D., Fink M., Shorter E., Wachtel L. E. (2011). Anti-NMDA receptor encephalitis versus pediatric catatonia. *The American Journal of Psychiatry*.

[31] Granata T., Matricardi S., Ragona F. (2018). Pediatric NMDAR encephalitis: a single center observation study with a closer look at movement disorders. *European Journal of Paediatric Neurology*.

[32] Moussa T., Afzal K., Cooper J., Rosenberger R., Gerstle K., Wagner-Weiner L. (2019). Pediatric anti-NMDA receptor encephalitis with catatonia: treatment with electroconvulsive therapy. *Pediatric Rheumatology Online Journal*.

[33] Herken J., Pruss H. (2017). Red flags: clinical signs for identifying autoimmune encephalitis in psychiatric patients. *Frontiers in Psychiatry*.

[34] Ferrafiat V., Raffin M., Deiva K. (2017). Catatonia and autoimmune conditions in children and adolescents: should we consider a therapeutic challenge?. *Journal of Child and Adolescent Psychopharmacology*.

[35] Alao A. O., Chlebowski S., Chung C. (2009). Neuropsychiatric systemic lupus erythematosus presenting as bipolar I disorder with catatonic features. *Psychosomatics*.

[36] Ditmore B. G., Malek-Ahmadi P., Mills D. M., Weddige R. L. (1992). Manic psychosis and catatonia stemming from systemic lupus erythematosus: response to ECT. *Convulsive Therapy*.

[37] Brenton J. N., Goodkin H. P. (2016). Antibody-mediated autoimmune encephalitis in childhood. *Pediatric Neurology*.

[38] Chandra S. R., Issac T. G., Shivaram S. (2015). Catatonia in children following systemic illness. *Indian Journal of Psychological Medicine*.

[39] Consoli A., Raffin M., Laurent C. (2012). Medical and developmental risk factors of catatonia in children and adolescents: a prospective case-control study. *Schizophrenia Research*.

[40] Denysenko L., Sica N., Penders T. M. (2018). Catatonia in the medically ill: etiology, diagnosis, and treatment. The academy of consultation-liaison psychiatry evidence-based medicine subcommittee monograph. *Annals of Clinical Psychiatry*.

[41] Duncan-Azadi C. R., Johnson P. N., Gormley A. (2017). Case report of midazolam withdrawal-induced catatonia in a 9-year-old patient. *A & A Case Reports*.

[42] Heekin R. D., Bradshaw K., Calarge C. A. (2019). First known case of catatonia due to cyclosporine A-related neurotoxicity in a pediatric patient with steroid-resistant nephrotic syndrome. *BMC Psychiatry*.

[43] Bilbily J., McCollum B., de Leon J. (2017). Catatonia secondary to sudden clozapine withdrawal: a case with three repeated episodes and a literature review. *Case Reports in Psychiatry*.

[44] Poser H. M., Trutia A. E. (2015). Treatment of a Prader-Willi patient with recurrent catatonia. *Case Reports in Psychiatry*.

[45] Bush G., Fink M., Petrides G., Dowling F., Francis A. (1996). Catatonia. II. Treatment with lorazepam and electroconvulsive therapy. *Acta Psychiatrica Scandinavica*.

[46] Petrides G., Divadeenam K. M., Bush G., Francis A. (1997). Synergism of lorazepam and electroconvulsive therapy in the treatment of catatonia. *Biological Psychiatry*.

[47] Pruett J. R., Rizvi S. T. (2005). A16-year-old girl with excited catatonia treated with low-dose oral lorazepam. *Journal of Child and Adolescent Psychopharmacology*.

[48] Cohen D., Flament M., Taieb O., Thompson C., Basquin M. (2000). Electroconvulsive therapy in adolescence. *European Child & Adolescent Psychiatry*.

[49] Consoli A., Benmiloud M., Wachtel L., Dhossche D., Cohen D., Bonnot O. (2010). Electroconvulsive therapy in adolescents with the catatonia syndrome. *The Journal of ECT*.

[50] Dhossche D. M., Withane N. (2019). Electroconvulsive therapy for catatonia in children and adolescents. *Child and Adolescent Psychiatric Clinics of North America*.

[51] Lee A., Glick D. B., Dinwiddie S. H. (2006). Electroconvulsive therapy in a pediatric patient with malignant catatonia and paraneoplastic limbic encephalitis. *The Journal of ECT*.

[52] Wachtel L. E., Crawford T. O., Dhossche D. M., Reti I. M. (2010). Electroconvulsive therapy for pediatric malignant catatonia with cerebellar dysgenesis. *Pediatric Neurology*.

[53] Wachtel L. E., Hermida A., Dhossche D. M. (2010). Maintenance electroconvulsive therapy in autistic catatonia: a case series review. *Progress in Neuro-Psychopharmacology & Biological Psychiatry*.

[54] Weiss M., Allan B., Greenaway M. (2012). Treatment of catatonia with electroconvulsive therapy in adolescents. *Journal of Child and Adolescent Psychopharmacology*.

[55] Yeung P. P., Milstein R. M., Daniels D. C., Bowers M. B. (1996). ECT for lorazepam-refractory catatonia. *Convulsive Therapy*.

[56] Zaw F. K. (2006). ECT and the youth: catatonia in context. *International Review of Neurobiology*.

[57] Zaw F. K., Bates G. D., Murali V., Bentham P. (1999). Catatonia, autism, and ECT. *Developmental Medicine and Child Neurology*.

[58] Wachtel L. E., Dhossche D. M., Kellner C. H. (2011). When is electroconvulsive therapy appropriate for children and adolescents?. *Medical Hypotheses*.

[59] Askenazy F., Dor E., Benoit M. (2010). Catatonia in a 14 year-old girl: treatment with clorazepam and carbamazepine, a 10-year follow-up. *Encephale*.

[60] Sharma C. M., Jena S., Sharma D., Agrawal R. P. (2014). Role of lorazepam challenge test in childhood catatonia. *Journal of Pediatric Neurosciences*.

[61] Huang Y. C., Lin C. C., Hung Y. Y., Huang T. L. (2013). Rapid relief of catatonia in mood disorder by lorazepam and diazepam. *Biomedical Journal*.

[62] Wachtel L. E. (2019). Treatment of catatonia in autism spectrum disorders. *Acta Psychiatrica Scandinavica*.

[63] Spiegel D. R., Glad R., Smith M., Raja U., Wade R., Johnson K. (2019). A case of schizophrenia with catatonia resistant to lorazepam and olanzapine monotherapy but responsive to combination treatment: is it time to consider using select second-generation antipsychotics earlier in the treatment algorithm for this patient type?. *Clinical Neuropharmacology*.

[64] Hefter D., Topor C. E., Gass P., Hirjak D. (2019). Two sides of the same coin: a case report of first-episode catatonic syndrome in a high-functioning autism patient. *Frontiers in Psychiatry*.

[65] Lim C. T., Stern J. L. (2019). Challenges of managing a first episode of pediatric catatonia. *Schizophrenia Research*.

[66] Tariq M., Afridi M. I., Saleem D., Pirzada S. (2019). Catatonic schizophrenia: cases with possible genetic predisposition. *Cureus*.

